# Optimization of an air-liquid interface *in vitro* cell co-culture model to estimate the hazard of aerosol exposures

**DOI:** 10.1016/j.jaerosci.2020.105703

**Published:** 2021-03

**Authors:** Rui-Wen He, Hedwig M. Braakhuis, Rob J. Vandebriel, Yvonne C.M. Staal, Eric R. Gremmer, Paul H.B. Fokkens, Claudia Kemp, Jolanda Vermeulen, Remco H.S. Westerink, Flemming R. Cassee

**Affiliations:** a National Institute for Public Health and the Environment (RIVM), P.O. Box 1, 3720, BA, Bilthoven, the Netherlands; b Institute for Risk Assessment Sciences, Utrecht University, P.O. Box 80178, 3508, TD, Utrecht, the Netherlands

**Keywords:** Aerosol exposures, Co-culture, Epithelial cells, Macrophages, Air–liquid interface, Barrier function

## Abstract

Inhalation exposure to environmental and occupational aerosol contaminants is associated with many respiratory health problems. To realistically mimic long-term inhalation exposure for toxicity testing, lung epithelial cells need to maintained and exposed under air-liquid interface (ALI) conditions for a prolonged period of time. In addition, to study cellular responses to aerosol particles, lung epithelial cells have to be co-cultured with macrophages. To that aim, we evaluated human bronchial epithelial Calu-3, 16HBE14o- (16HBE), H292, and BEAS-2B cell lines with respect to epithelial morphology, barrier function and cell viability under prolonged ALI culture conditions. Only the Calu-3 cells can retain the monolayer structure and maintain a strong tight junction under long-term ALI culture at least up to 2 weeks. As such, Calu-3 cells were applied as the structural barrier to create co-culture models with human monocyte-derived macrophages (MDMs) and THP-1 derived macrophages (TDMs). Adhesion of macrophages onto the epithelial monolayer was allowed for 4 h with a density of 5 × 10^4^ macrophages/cm^2^. In comparison to the Calu-3 mono-culture model, Calu-3 + TDM and Calu-3 + MDM co-culture models showed an increased sensitivity in inflammatory responses to lipopolysaccharide (LPS) aerosol at Day 1 of co-culture, with the Calu-3 + MDM model giving a stronger response than Calu-3 + TDM. Therefore, the epithelial monolayer integrity and increased sensitivity make the Calu-3 + MDM co-culture model a preferred option for ALI exposure to inhaled aerosols for toxicity testing.

## Highlights

• Calu-3+MDM model shows the epithelial monolayer integrity under prolonged ALI culture.
• Calu-3+MDM model has an increased sensitivity in inflammatory responses to LPS aerosol.
• Combination of Calu-3+MDM model is a preferred option for long-term ALI exposure to inhaled aerosols.

## 1 Introduction

Humans constantly inhale various exogenous substances such as nanoparticles, traffic emissions, and cigarette smoking aerosols (Almstrand et al., 2009; Phillips et al., 1999). Inhalation exposure to environmental and occupational aerosol contaminants is related to a wide range of public health problems such as chronic respiratory diseases and airway dysfunction (Bakand et al., 2012; Samet & Krewski, 2007). These effects on respiratory health can be studied using *in vivo* and *in vitro* methods. Due to limitations of *in vivo* experiments including ethical issues, inter-species differences and operational difficulties, *in vitro* models have increasingly been applied for hazard assessment of aerosol exposures (BéruBé et al., 2010; Braakhuis et al., 2016). The commonly used *in vitro* approach involves dissolving the aerosols of interest in culture medium to expose lung cells under submerged conditions. However, submerged exposure conditions do not adequately resemble the *in vivo* situation, as under realistic conditions a gradual delivery and deposition from the air onto the respiratory tract lining the epithelium will occur (BeruBe et al., 2009). Besides, characteristics and kinetics of the test substances will likely change during submerged exposure. Therefore, the relevance of biological responses observed following submerged exposure has been debated (Limbach et al., 2005; Mülhopt et al., 2016).

To minimize these limitations, air-liquid interface (ALI) exposure of cells has been developed. ALI exposure of cells is applied by the exposure systems, which use a continuous flow or single cloud to expose cells to aerosols containing test substances, enabling a more relevant and realistic inhalation exposure (BéruBé et al., 2010; Mülhopt et al., 2016). A single layer of epithelial cells covers the surface of bronchioles, the first target of exposure to inhaled substances (BéruBé et al., 2010; Hiemstra et al., 2018). Consequently, the corresponding characteristics of epithelium such as single-layer morphology and barrier functions are regarded as essential criteria for cell selection at the ALI (Hermans & Bernard, 1999). For ALI conditions, cells are cultured on the apical side of membrane fitted in an insert, which is exposed to air to simulate human airway conditions (de Jong et al., 1994; Paur et al., 2011). Culture medium is added to the basolateral side of the insert to be in contact with cells for nutrient supply via the membrane. Such an approach requires careful selection of the appropriate cell types/lines, since not that many cell types can be cultured under ALI conditions and still remain viable for a sufficiently long period of time. While current epithelial cell models have been evaluated for their suitability for culture under ALI conditions for only a few days (Heijink et al., 2010), there is an increasing need to expose these cell models for multiple days to resemble repeated and even long-term exposure to inhaled aerosols (PATROLS-Website, 2018).

Macrophages, the most abundant immune-cell type present in healthy lungs, play an important role in the clearance of foreign substance and apoptotic cells (Hu & Christman, 2019; Septiadi et al., 2018). To mimic the lung epithelium more closely, co-culture models have been designed by adding macrophages on the top of the epithelial cell layer (Ji et al., 2018; Lehmann et al., 2011). These macrophages are intended to produce inflammatory responses that can affect epithelial cells, probably increasing the sensitivity of co-culture models to inhaled aerosol particles in comparison to mono-culture models (Tao & Kobzik, 2002; Wottrich et al., 2004). Due to easy handling and high reproducibility, macrophages differentiated from THP-1 human monocytes (THP-1 derived macrophages, TDMs) were mostly used in co-cultures (Chanput et al., 2014), whereas human monocyte-derived macrophages (MDMs) can provide more realistic cellular responses (Lehmann et al., 2011). However, information on the number of days that MDMs and TDMs are viable is currently lacking and it is unknown if they retain their functions in co-culture models.

We therefore evaluated widely-used epithelial cell models including 16HBE14o- (16HBE), Calu-3, H292 and BEAS-2B cells under prolonged ALI culture conditions in terms of their epithelial morphology, barrier function and cell viability. In addition, MDMs and TDMs were used to create co-culture models and to evaluate the number of days that they remained viable as well as their functional responses to LPS aerosol.

## 2 Materials and methods

### 2.1 Cell cultures

16HBE, Calu-3, H292 and BEAS-2B cells are widely used as lung epithelial cell models in submerged and ALI culture (see the supplementary information for additional details). 16HBE cells were kindly provided by Dr. Gruenert (University of California, San Francisco, CA). Calu-3, H292 and BEAS-2B cells were purchased from the American Tissue Culture Collection (ATCC, Rockville, MD). 16HBE cells (passage 12–18) were cultured in Dulbecco's Modified Eagle Medium (DMEM)/F-12 supplemented with 1% l-Glutamine, 1% Fungizone, 5% Fetal Bovine Serum (FBS), and 1% Penicillin-Streptomycin (P–S); Calu-3 cells (passage 05–12) were cultured in minimum essential medium (MEM) with 10% FBS, 1% Non-Essential Amino Acid (NEAA) solution and 1% P–S; H292 cells (passage 05–09) were cultured in RPMI-1640 medium with 10% FBS, 1% P–S and 1% sodium pyruvate; BEAS-2B cells (passage 05–09) were cultured in DMEM medium with 10% FBS and 1% P–S.

THP-1 monocyte-like cells (ATCC, Rockville, MD) and primary human CD14^+^ monocytes isolated from buffy coats (Sanquin, Amsterdam, the Netherlands) were differentiated to macrophages. THP-1 cells (passage 08–13) were differentiated to TDMs by addition of phorbol 12-myristate 13-acetate (PMA, 30 ng/mL, Sigma, the Netherlands) for 5 days, followed by culturing with fresh medium for two more days. Primary human CD14^+^ monocytes were differentiated to MDMs by addition of macrophage colony-stimulating factor (M-CSF, 50 ng/mL, Sigma, the Netherlands) for 6 days (Lehmann et al., 2011). Monocytes and macrophages were cultured in RPMI-1640 medium with 10% FBS and 1% P–S. To avoid the variation between donors, the isolated primary human CD14^+^ monocytes were frozen in separate tubes until use to ensure MDMs used in this study were from the same donor. Cells were cultured in the flask in an incubator at 37 °C with 5% CO_2_. All culture medium and supplements were purchased from Life Technologies (Thermo Fisher Scientific Inc., the Netherlands).

### 2.2 Preparation of air-liquid interface (ALI) culture

**Fig. 1:**
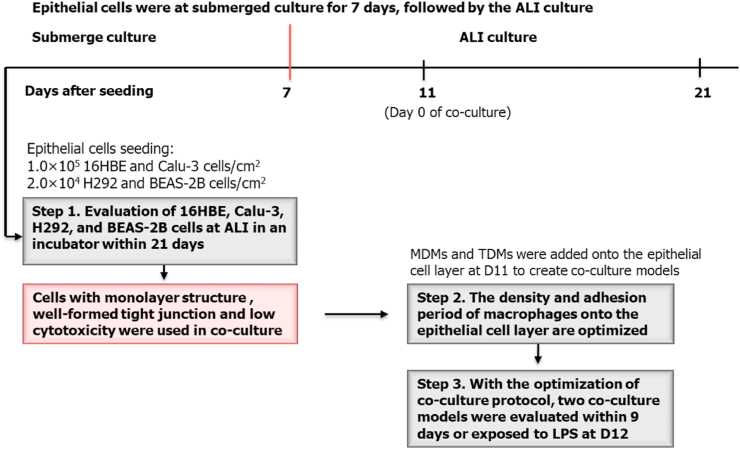
A schematic overview of the procedures including cell seeding, creating co-culture models and LPS exposure for the epithelial mono-culture and macrophage/epithelial cell co-culture models. Epithelial cells were at submerged culture for 7 days, followed by ALI culture.

A schematic overview on the cell seeding and exposure is shown in Fig. 1. When reaching approximately 80% confluency in the cell culture flask, 16HBE, Calu-3, H292, and BEAS-2B cells were detached enzymatically (0.05% trypsin-EDTA, Thermo Fisher Scientific Inc., the Netherlands) and subsequently seeded on the apical side of inserts (0.4 μm pore membrane, Polyester, Corning Inc., Germany) fitted in a 6- or 12- wells plate with respectively 1 or 0.5 mL corresponding culture medium on the apical side and 2 or 1.5 mL culture medium was added into the basolateral side. The submerged culture period to reach confluence was set at 7 days. H292 and BEAS-2B cells grow much faster in submerged conditions compared to 16HBE and Calu-3 cells. To prevent overgrowing before ALI culture, seeding densities were set at 1.0 × 10^5^ cells/cm^2^ for 16HBE and Calu-3 cells, and 2.0 × 10^4^ cells/cm^2^ for H292 and BEAS-2B cells. After submerged culture for 7 days, apical medium was removed from the inserts to obtain the ALI conditions. Morphology of the epithelial cell models in the inserts was monitored over time via an optical microscope (OM) (CKX41 Inverted Microscope, Olympus, Waltham, USA) with a 4-time objective lens.

To create co-culture models, macrophages were added onto the epithelial cell layer at the fourth day of ALI culture, which was set as day 0 (D0) for co-culture. MDMs were gently scraped from the flask with cell dissociation buffer (Thermo Fisher Scientific Inc., the Netherlands), while TDMs were detached enzymatically with Accutase (Thermo Fisher Scientific Inc., the Netherlands) due to their low cell viability after scraping. After re-suspension, 1 or 0.5 mL of macrophage suspension (2.0 × 10^4^ or 5 × 10^4^ macrophages/cm^2^, some with labelling as described in 2.5) was added onto the epithelial cell layer in 6- or 12 -wells inserts to allow the adhesion of macrophages. In our pilot experiments we used a minimum of 2 h for macrophages adhesion. However, microscopic observation showed that the majority of the cells was not able to adhere to the epithelial carpet within 2 h of adhesion. We therefore increased the adhesion time from 2 h to 4 h. To evaluate the influence of re-added apical medium on the morphology of the cell models and the adhesion efficiency of the macrophages, we prolonged the adhesion time to 24 h. From a practical point of view, we did not select an adhesion time between 4 and 24 h, as it will make the removal of apical medium as well as the following ALI exposure less convenient. The Calu-3 mono-culture model was also treated using same protocol, but adding 1 or 0.5 mL of culture medium. Mono- and co-culture models in 6-well inserts were evaluated over the co-culture time while in 12-well inserts the cells were exposed to LPS aerosol at D1 of the co-culture.

For all cell models in inserts, apical and basolateral medium was refreshed every 2–3 days and collected separately. To collect the apical medium at the ALI for measuring LDH and cytokine release (see 2.7), 1 and 0.5 mL of corresponding medium was added to the apical side of 6- and 12 -wells inserts respectively, incubated for around 30 min, and harvested.

### 2.3 Lipopolysaccharide (LPS) aerosol exposure

LPS (Thermo Fisher Scientific Inc., the Netherlands) as a positive control substance was sprayed onto the apical side of mono- and co-culture models in 12 well inserts via the VITROCELL® cloud exposure system (Fig. S1, Vitrocell, Waldkirch, Germany) at D1 of the co-culture. The injection volume of LPS solution (175 μg/mL) for nebulization was 200 μL and the deposited dose in each insert was 0.25 μg/cm^2^ as measured using a microbalance. The LPS exposure in the Cloud exposure system takes about 15 min 3–4 inserts were used for LPS exposure in Calu-3 mono-culture, Calu-3 + MDM, and Calu-3 + TDM models; Due to the similar cellular responses between air control and incubator control (Fig. S7) (He et al., 2020), we chose to place 3 inserts under ALI conditions in an incubator as control. Apical and basolateral medium were collected separately (described in 2.2) after exposure for 24 h.

### 2.4 Transepithelial electrical resistance (TEER) measurement

As an important indicator of barrier integrity, transepithelial electrical resistance (TEER) was measured using an Evom2 Voltohmmeter equipped with 4 mm chopstick electrodes (World Precision Instruments Inc., FL, USA). To measure TEER at the ALI, 1 mL of corresponding medium was added onto the apical side of a 6-well insert. All TEER values were corrected for the resistance of cell-free insert (≈130 Ω) and the surface area of a 6-well insert (4.67 cm^2^).

### 2.5 Zonula occludens protein-1 (ZO-1) staining

Tight junctions play a crucial role in epithelial barrier function, therefore cultures were stained for the tight-junction protein ZO-1 in two ways, depending on the microscope used and research aim. To visualize tight junctions and the monolayer structure of epithelial cells, method 1 was used with confocal laser scanning fluorescence microscopy (CLSM); to visualize the epithelial cell layer in Calu-3 mono-culture and co-culture models, method 2 was used with fluorescence microscopy (FM). Cells were washed 3 times with phosphate buffered saline (PBS) and fixed in 4% paraformaldehyde (Thermo Fisher Scientific Inc., the Netherlands) for 5 min, then permeabilized with 0.1% Triton X-100 (Thermo Fisher Scientific Inc., the Netherlands) for 15 min.

Method 1: cells were incubated with the ZO-1 rabbit polyclonal antibody (Thermo Fisher Scientific, Switzerland, 1:100 in 0.1% bovine serum albumin (BSA) in PBS) for 2 h, followed by another 2 h incubation with a mixture of secondary antibodies in 0.1% BSA in PBS: goat anti-rabbit immunoglobulin G (IgG) DyLight 488 conjugated (Agrisera, Sweden, 1:100 dilution), rhodamine-phalloidin (Thermo Fisher Scientific, Switzerland, 1:100 dilution) and 1 μg/mL 4′ 6-diamidino-2-phenylindole (DAPI) (Sigma Aldrich, Switzerland). All the staining steps were performed in the dark at room temperature. After staining, the cells were washed with PBS, and mounted in glycergel (DAKO Schweiz AG, Switzerland) in microscopy slides, subsequently visualized via CLSM (Carl Zeiss, Switzerland) equipped with a 40-times objective lens.
Method 2: cells were incubated with the ZO1-1A12 monoclonal antibody (Thermo Fisher Scientific Inc., the Netherlands, 1:500 in 0.2% Triton X-100) for 30 min, followed by another 30 min incubation in the dark with a secondary antibody, fluorescein FITC anti-mouse IgG (Thermo Fisher Scientific Inc., the Netherlands, 1:100 in PBS). Cell nuclei were counter-stained afterwards by 1 μg/mL DAPI (Thermo Fisher Scientific Inc., the Netherlands) in PBS for 7 min. In between all steps, cells were washed 2 or 3 times with PBS. Culture inserts were carefully mounted on a microscope slide with a glycerol-based liquid (Thermo Fisher Scientific Inc., the Netherlands) and examined via FM (Olympus BX51, Shinjuku, Japan) with a 10-time objective lens.

### 2.6 Macrophages morphology

Before adding onto the epithelial carpet, the morphology of MDMs and TDMs was assessed via the May Grünwald Giemsa stain. Briefly, macrophages were stained with May-Grünwald solution (Merck, Germany) for 5 min on slides. After rinsing with Milli-Q water, macrophages were subsequently stained with 0.8% (V/V) Giemsa solution in Sorensen's phosphate buffer (Thermo Fisher Scientific Inc., the Netherlands) for 20 min. Afterwards, slides were rinsed thoroughly with Milli-Q water and air dried, which were then assessed with an OM (Leitz Laborlux D, Leica, Germany) at a 50-time objective lens.

To visualize macrophages in the co-culture models, macrophages were pre-labelled with Vybrant DiI dye (Thermo Fisher Scientific Inc., the Netherlands) according to Septiadi et al. (2018). After detachment from the flask, macrophages (10^5^ macrophages/mL < density <10^7^ macrophages/mL) were incubated in the dye-containing medium (dye: medium = 1:200, v/v) for approximately 25 min, followed by washing 3 times with culture medium. By counting cells under the OM and FM, the dye labelling efficiency of macrophages was calculated:

$$\text{Dye}\;\text{labeling}\;\text{efficiency}\left(\%\right)=\frac{Macrophages\;number\;under\;FM}{Macrophages\;number\;under\;OM}\times 100\%$$

As described in 2.2, the labelled macrophages were added onto the epithelial cell layer (2 × 10^4^ macrophages/cm^2^). After adhesion of macrophages for 4 or 24 h, inserts were examined under the FM, with 4–6 images of macrophages being taken at random areas of the membrane. By counting the number of macrophages in each image, the efficiency of macrophages adhesion can be calculated as follows:

$$\text{Adhesion}\;\text{efficiency}\left(\%\right)=\frac{Macrophages\;number\;in\;image\times \frac{4.67}{0.0059\;}}{Total\;adding\;number\times Dye\;labeling\;efficiency}\times 100\%$$

in which the correction from image area (0.59 mm^2^) to surface area of the 6-well insert (4.67 cm^2^) is included.

### 2.7 Cell viability, LDH release and inflammatory cytokine release

The MTS assay was used to test the cell viability (Promega, Fitchburg, Wisconsin, USA). Briefly, cells on the apical side of the inserts were incubated with the MTS solution (medium: MTS reagent = 9: 1, v/v) for 60 min before absorbance measurement by a microplate reader (SpectraMax M2: Molecular Devices, Sunnyvale CA, USA). Values of cell viability were corrected for the incubator controls.

To study cytotoxicity and cell membrane integrity, lactate dehydrogenase (LDH) release in the apical and basolateral medium was measured (Roche Diagnostics GmbH, Mannheim, Germany). Briefly, 100 μL of supernatant and 100 μL reaction reagent were successively added into a 96-well flat-bottomed plate and incubated in the dark for 20 min at room temperature. After adding 50 μL stop solution (HCl, 1.0 M, Sigma, Netherlands) per well, the absorbance was measured. All LDH values were corrected for the maximum LDH release per cell type or for the incubator controls. To measure the maximum LDH release, cells were incubated with lysis buffer (2% Triton X-100, Thermo Fisher Scientific Inc., the Netherlands) for 5 min. The lysate was collected for LDH measurement.

To test the inflammatory response, inflammatory cytokines (interleukin (IL)-1β, IL-8, IL-10 and tumor necrosis factor (TNF)-α) in the apical and basolateral medium were measured using an enzyme-linked immunosorbent assay (ELISA) (Thermo Fisher Scientific Inc., the Netherlands) according to the manufacturer's protocol.

### 2.8 Statistical analysis

Results from mono-culture models were obtained from two independent experiments, with 3–6 parallel inserts/supernatants in each experiment; results from co-culturing with macrophages were obtained from one experiment, with 6–12 parallel inserts/supernatant. Results from LPS exposure were obtained from one experiment, with 3–4 parallel inserts. Error bars indicate standard deviation of the mean (SD). Differences between groups were compared by one-way analysis of variance (ANOVA), a p-value ≤ 0.05 is considered statistically significant. Data analysis was conducted using GraphPad software (version 8.2.1).

## 3 Results

### 3.1 Mono-culture models at the ALI

**Fig. 2:**
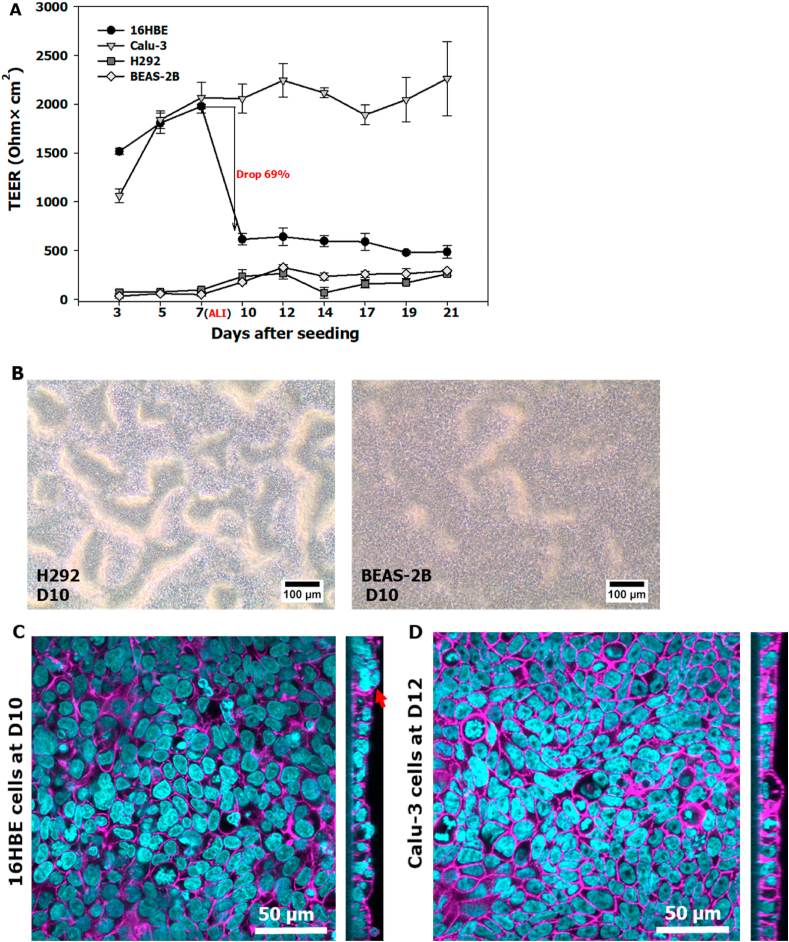
TEER (A) of 16HBE, Calu-3, H292 and BEAS-2B cells during the 21-day culture period, cells were at submerged culture for 7 days, followed by ALI culture; error bars indicate the standard deviation of 6 parallel inserts with cells. Cell morphology (B) of H292 and BEAS-2B cells at Day 10; Confocal fluorescence microscopy images of 16HBE cells at Day 10 (C) and Calu-3 cells at Day 12 (D); left: XY viewing direction; right: Z-stack section. Tight junction ZO-1 proteins (C and D) were stained in purple and the nuclei in turquoise. Multilayers in 16HBE cells at D10 (red arrow). Scale bars in B: 100 μm; in C and D:50 μm. (For interpretation of the references to colour in this figure legend, the reader is referred to the Web version of this article.)

TEER changes of 16HBE, Calu-3, H292 and BEAS-2B cells were followed and their morphology was assessed over the 21-day culture period, and tight junctions of 16 HBE cells (D10) and Calu-3 cells (D12) were visualized with ZO-1 staining method 1 (Fig. 2). In line with previous studies (Chen et al., 2015; Srinivasan et al., 2015), we classified barrier function of respiratory epithelial cell models in 6-well insert as “tight” with TEER values higher than 1000 Ω × cm^2^, as “intermediate” with values between 300 and 1000 Ω × cm^2^, and as “leaky” with values below 300 Ω × cm^2^. During submerged culture, 16HBE and Calu-3 cells showed similar trends of increasing TEER values over time, reaching around 2000 Ω × cm^2^ (Fig. 2A). When changing to ALI conditions, TEER values for 16HBE cells dropped 69% to around 550 Ω × cm^2^ with the functional network of tight junction ZO-1 protein (stained with method 1) incompletely defined (Fig. 2C, XY direction), while values for Calu-3 cells fluctuated around 2000 Ω × cm^2^ with well-defined ZO-1 proteins expression around the cell periphery (Fig. 2D, XY direction). H292 and BEAS-2B cells showed low TEER values (around 300 Ω × cm^2^) in both culture conditions during the whole culture period.

After submerged culture for 7 days, confluent cell monolayers were seen for all cell lines (Fig. S2A). However, H292 and BEAS-2B cells appeared as multilayers within 3 days culture at the ALI (D10, Fig. 2B), which became more obvious at D12 (Fig. S2B). A small scale of multilayers was also observed in 16HBE cells at D10 (Fig. 2C, Z-stack section), indicating the loss of monolayer character. Only Calu-3 cells kept their monolayer structure (Fig. 2D, Z-stack section) during long-term ALI culture (≈2 weeks) that can be seen up to 5 weeks (Fig. S2C).

**Fig. 3:**
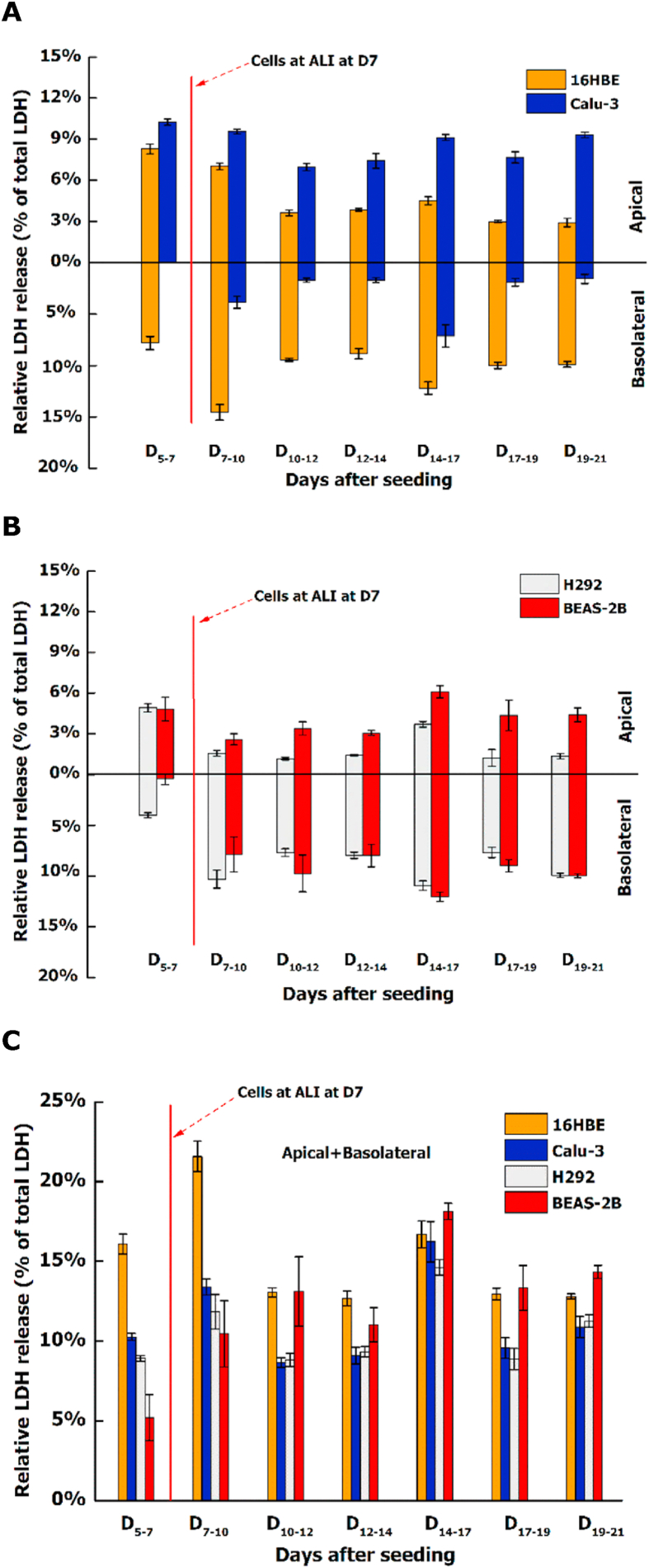
Relative LDH release on the apical (A) and basolateral (B) side of 16HBE, Calu-3, H292 and BEAS-2B cells as well as their total LDH levels (C) during the 21-day culture period. LDH release was measured during 2 or 3 days. The total LDH release was calculated by adding the relative LDH levels on both sides. Error bars indicate the standard deviation of 5 or 6 parallel inserts with cells.

Relative LDH release (corrected for the maximum LDH release) in the apical and basolateral medium was measured every 2 or 3 days, and the total LDH release was also calculated by adding the relative LDH levels on both sides (Fig. 3). Overall, total LDH levels showed a slight increase for all cell lines when changing submerged culture conditions to the ALI, followed by fluctuations around 13% for 16HBE and BEAS-2B cells, and around 9% for Calu-3 and H292 cells. Among these cell lines, Calu-3 cells showed the highest LDH release (≈7%) in the apical medium and the lowest LDH release (≈2%) in the basolateral medium.

Due to the inability to develop a significant TEER, the occurrence of obvious multilayer structure and relatively higher LDH levels under ALI conditions, 16HBE, H292 and BEAS-2B cells were disregarded for additional experiments in this study, whereas Calu-3 culture model was used for subsequent co-cultures experiments.

### 3.2 Creating co-culture models

Before co-culture, MDMs and TDMs were stained in chamber slides to characterize morphology. As shown in Fig. S3, the round and oval nuclei were peripherally and centrally located in macrophages, with the larger nuclei in TDMs. Some of MDMs and TDMs developed irregular cytoplasm and pseudopodia, suggesting differentiation of macrophages (Vordenbäumen et al., 2013).

**Fig. 4:**
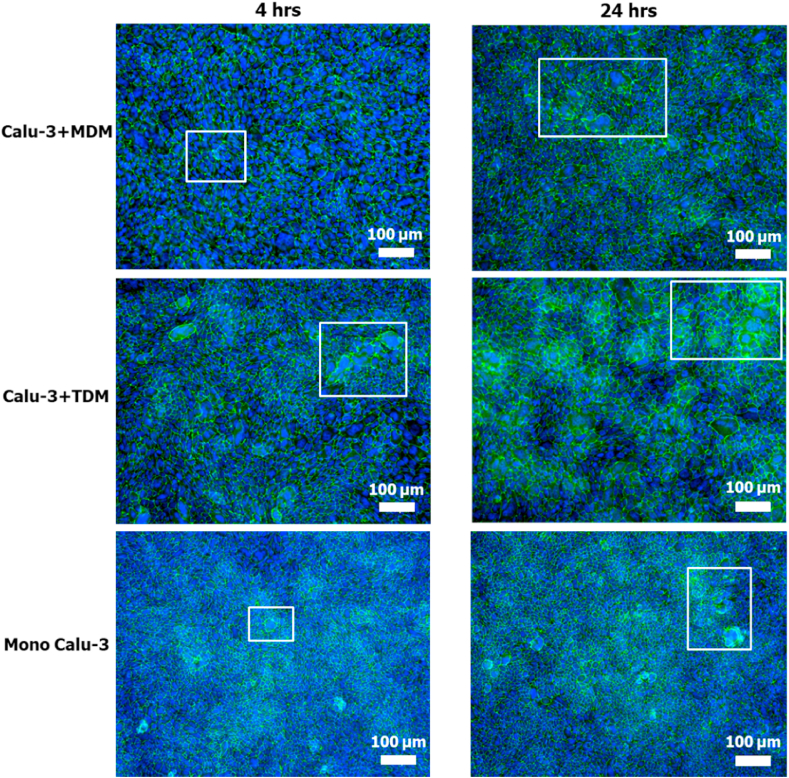
Fluorescence microscopy images of epithelial cell layer in mono-culture Calu-3 and co-culture models after 4 (left) and 24 h (right) adhesion period. The tight junction ZO-1 proteins were stained in green and nuclei in blue. White squares are examples of cell fusion. Scale bars: 100 μm. (For interpretation of the references to colour in this figure legend, the reader is referred to the Web version of this article.)

**Fig. 5:**
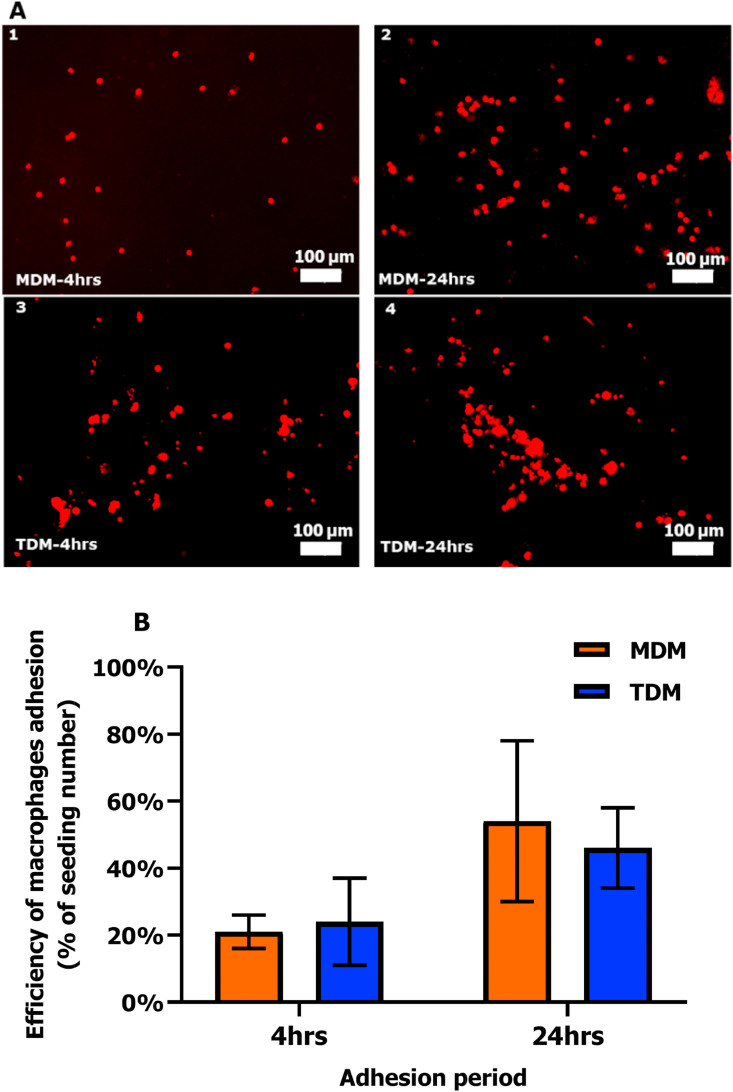
Fluorescence microscopy images (A) of MDMs and TDMs (2.0 × 10^4^ cells/cm^2^) after 4 (left) and 24 h (right) adhesion onto the Calu-3 epithelial carpet and their adhesion efficiency (B). Macrophages were labelled with Vybrant DiI dye (in red, A). Scale bars in A: 100 μm. B: Error bars indicate the standard deviation of macrophages number in 4 or 6 random areas of the insert membrane. More images of MDMs and TDMs in Fig. S4. (For interpretation of the references to colour in this figure legend, the reader is referred to the Web version of this article.)

After culturing with macrophages for 4 or 24 h (adhesion period), tight junctions of the epithelial cell layer were visualized (Fig. 4, ZO-1 staining method 2, see 2.5). The strong ZO-1 expression around the cell periphery was observed in mono- and co-culture models, while cell fusions occurred as well, leading to the formation of some large cells with multiple nuclei or a single larger nucleus. Compared to 4 h, cell fusions appeared at a larger scale in mono- and co-culture models with 24 h adhesion period, resulting in the formation of multilayers of cells, in particular for the Calu-3+TDM model. The number of MDMs and TDMs on the epithelial cell layer as well as their adhesion efficiency were also related to the adhesion time periods (Fig. 5 and Fig. S4). Overall, the larger number of adherent macrophages was seen in the co-culture models after 24 h adhesion, resulting in a higher adhesion efficiency (≈55%) of MDMs and TDMs in comparison to their efficiency after 4 h (≈20%).

To avoid the large scale formation of multilayers in the co-culture models, 4 h adhesion of macrophages was used for creating the co-culture models. In our pilot study no IL-1β, an inflammatory indicator for macrophages, was detected in the co-culture models in response to LPS aerosol when using a density of 2.0 × 10^4^ macrophages/cm^2^. Since the number of adherent macrophages can influence the sensitivity of co-culture models (Bodet et al., 2005), we increased the seeding density from 2 × 10^4^ to 5 × 10^4^ macrophages/cm^2^ to enhance the sensitivity of the co-culture models. The adherence efficiency of MDMs and TDMs at a seeding density of 5 × 10^4^ macrophages/cm^2^ after 4 h was 17% ± 9% and 13% ± 7%, respectively, which was rather similar with their efficiency at a seeding density of 2 × 10^4^ macrophages/cm^2^.

### 3.3 Co-culture models at the ALI

**Fig. 6:**
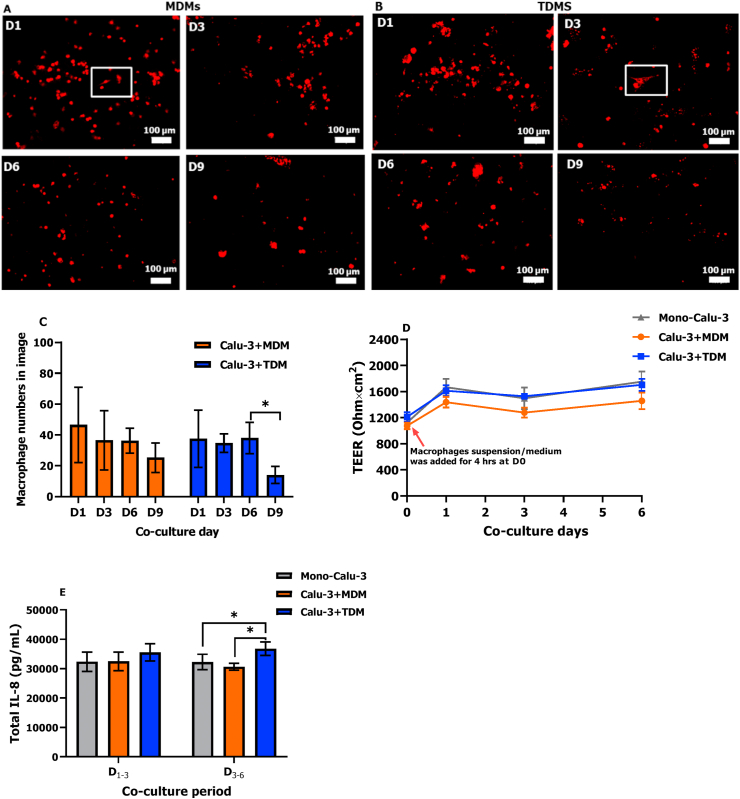
Fluorescence microscopy images of MDMs (A) and TDMs (B) (4 h adhesion, density: 5.0 × 10^4^ cells/cm^2^) and their average number in images (C) after co-culture for 1, 3, 6, and 9 days at the ALI, as well as the TEER (D) and IL-8 production (E) during the 6-day co-culture period. White squares in (A) and (B) are morphological examples of the MDMs and TDMs differentiation. Scale bars in (A) and (B): 100 μm. Error bars in (C) indicate the standard deviation of macrophage numbers in 3 or 4 random areas of the insert membrane; Error bars in (D) and (E) indicate the standard deviation of 6 or 12 parallel inserts with cells. * represents p < 0.05.

To determine the number of days that macrophages remained viable, morphology of MDMs and TDMs on the epithelial carpet was assessed during the 9-day co-culture period (Fig. 6A and B). Overall, MDMs and TDMs showed similar morphological changes. At D1 and D3 of the co-culture, morphological differentiation was seen in MDMs and TDMs that formed aggregates and developed pseudopodia. During the 6 days co-culture, the numbers of macrophages as well as Calu-3 cells were similar at Day 1, 3, and 6 (Fig. 6C and Table S1), suggesting that the ratio between Calu-3 cells and macrophages in our co-culture models remained stable (≈20). However, increased apoptotic cell debris appeared after a longer period of the co-culture (D6 and D9) with a drop in macrophage numbers at D9 (Fig. 6C), indicating that more macrophages, particularly TDMs, had died after the long culture period at the ALI (>6 days).

To evaluate the responses of co-culture models to ALI conditions, TEER changes and total IL-8 and IL-1β release in mono- and co-culture models after two time periods (D_1-3_ and D_3-6_) were measured (Fig. 6D and E). All cell models showed a comparable increase in TEER values from D0 to D1 that resulted in a level of around 1500 Ω × cm^2^ (Fig. 6D). IL-1β could not be detected in mono- and co-culture models after each time period (data not shown), while IL-8 could be detected in each cell model and showed no difference between D_1-3_ and D_3-6_ (Fig. 6E). All cell models showed a comparable IL-8 level at D_1-3_, while a significant increase (p < 0.05) was seen in the Calu-3+TDM model at D_3-6_ in comparison to the mono-culture and Calu-3 + MDM models.

### 3.4 Functional responses of co-culture models to LPS aerosol

**Fig. 7:**
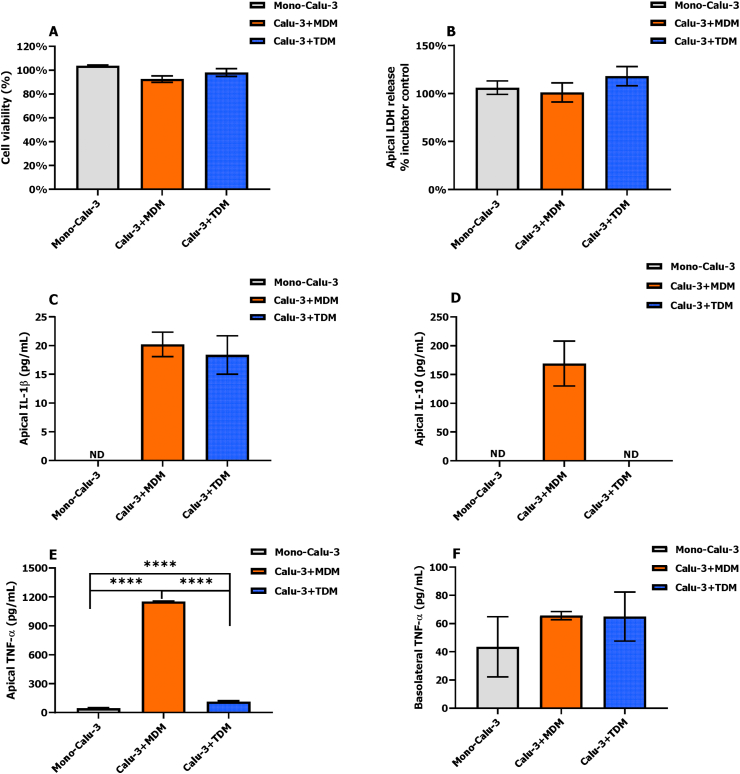
Effects of LPS exposure in mono- and co-culture models in 12 well inserts including cell viability (A), LDH release (B) and the production of IL-1β (C), IL-10 (D) and TNF-α (E) on the apical side and TNF-α (F) on the basolateral side. Error bars indicate the standard deviation of 3 or 4 parallel inserts with cells. ND represents not detected; **** represents p < 0.0001.

Cell viability, LDH release and production of the inflammatory cytokines IL-1β, IL-10 and TNF-α in mono- and co-culture models were measured after LPS exposure (D1 of the co-culture) for 24 h (Fig. 7). All cell models showed high cell viability (>90%) and no changes in LDH release upon LPS exposure (Fig. 7A and B), indicating no cytotoxicity. No effect of LPS on the release of IL-1β and IL-10 was seen in the mono-culture model. A comparable level of IL-1β was detected on the apical side of both co-culture models, while IL-10 was only detected on the apical side of the Calu-3 + MDM model (≈150 pg/mL) (Fig. 7C and D). TNF-α that can be produced by both macrophages and Calu-3 cells was detected on both sides of all cell models (Fig. 7E and F). On the apical side, the Calu-3 + MDM model showed the highest concentration of TNF-α (≈1150 pg/mL, p < 0.0001), followed by the Calu-3 + TDM model (≈115 pg/mL), which was significantly higher than the concentration in the mono-culture (≈45 pg/mL, p < 0.0001). No significant difference (p > 0.05) was seen in the TNF-α levels on the basolateral side of all cell models.

For all cell models, no LDH was detected on the basolateral side of the incubator controls and exposed samples; IL-1β, IL-10 and TNF-α were not detected on either side of the incubator controls, and also no IL-1β and IL-10 on the basolateral side of exposed samples.

## 4 Discussion

Our results demonstrate the substantial differences in cellular responses including cell morphology, TEER changes, and cytotoxicity for epithelial mono-culture of 16HBE, Calu-3, H292 and BEAS-2B cells as well as the macrophage/epithelial cell co-culture models under ALI conditions. The Calu-3 epithelial model can retain its monolayer structure and develop a strong tight junction under long-term ALI culture, which was subsequently used as the structural barrier to create co-culture models in combination with MDMs and TDMs. With the optimization of the co-culture procedure at the ALI, our cell co-culture models showed epithelial monolayer integrity, and increased sensitivity in inflammatory responses to LPS exposure, with the Calu-3 + MDM model giving the strongest responses.

### 4.1 Mono-culture models at the ALI

Human bronchial epithelial and alveolar epithelial cell models are both viable options for *in vitro* exposure, especially to ultrafine particles (UFPs), since both the tracheobronchial and alveolar regions will receive UFPs on their epithelial surface. Deposition of UFPs is not limited to the alveoli, and with decreasing air velocity, substantial deposition can also occur on the terminal bronchial epithelium (Braakhuis et al., 2014). Therefore, human bronchial epithelial cell lines such as 16HBE, Calu-3, H292 and BEAS-2B cells are suitable lung models to study inhalation exposure (BéruBé et al., 2010; Hiemstra et al., 2018). Primary bronchial epithelial cells that can be cultured at the ALI for weeks with well-formed tight junctions might be a good alternative to the lung epithelial cell lines (Pezzulo et al., 2011). However, they have some disadvantages including the high cost, difficult handling procedures and donor variations, which make them less suitable as a basis for a co-culture model from an economical, reproducible and practical point of view. Indeed, to comprehensively evaluate inhalation toxicity of, for example, UFPs *in vitro*, it is warranted to study their effects both in bronchial epithelial models and alveolar epithelial cell models. Therefore, further investigation on which is the best alveolar model is also needed.

When culturing epithelial cell models at the ALI, we assessed the formation of monolayer structure and barrier function as markers of the ability to resist the transport of xenobiotics (BéruBé et al., 2010; Holgate, 2008; Kidney & Proud, 2000). According to our findings, Calu-3 epithelial cells can act as basis for the co-culture models, since they can develop a strong barrier function and keep their monolayer structure for long-term ALI culture with low cytotoxicity. Depending on the surface and pore size of insert membrane, culture period and medium refreshment frequency, TEER values of Calu-3 cells can differ widely varying from 100 to 2500 Ω × cm^2^ (Srinivasan et al., 2015). In this study, we set TEER values > 1000 Ω × cm^2^ in a 6-well insert before starting the co-culture as the criterion for the Calu-3 cell model. When forming a high barrier integrity at the ALI, the permeability coefficient of compounds across the Calu-3 monolayer would decrease with increasing molecular weight (Grainger et al., 2006). This might be an explanation for the observation that LDH (≈140 kDa) accumulated on the apical side of the Calu-3 monolayer, while the level of LDH detected in the basolateral medium was only limited (Fig. 3). In comparison, for 16HBE, H292 and BEAS-2B cells with a weak barrier function, the LDH level on the basolateral side was generally higher than that on the apical side. A similar finding was also reported for A549 cells that lack functional tight junctions at the ALI and showed higher LDH levels in basolateral than in apical medium after exposure to polluted air (Zavala et al., 2016). These findings indicate that LDH has a high permeability across cell layers with a weak barrier function, therefore LDH levels in the basolateral medium could be an indicator to evaluate the barrier function of cells at the ALI. Also when evaluating and comparing the cytotoxicity at the ALI, LDH levels on the apical and basolateral side should be considered respectively depending on the barrier function of cells.

### 4.2 Co-culture models at the ALI

The beneficial properties of the Calu-3 epithelial monolayer barrier can be increased by addition of pulmonary macrophages, which play an important role in the defence of airways (Byrne et al., 2015). In the present study, improvements were made by adding MDMs and TDMs onto the Calu-3 monolayer to create the co-culture models. However, there is no consensus regarding the optimal number of macrophages and their adhesion time to the epithelial cell layer. The commonly used seeding ratio between macrophages and epithelial cells ranges from 1:1 to 1:5 with an adhesion time from 2 h to 24 h (Bodet et al., 2005; Grabowski et al., 2016; Lehmann et al., 2011; Tao & Kobzik, 2002; Ugonna et al., 2014). Our results demonstrate that a long time period (e.g. 24 hrs) with medium on top of the monolayer can affect its structure. To avoid this negative effect on the monolayer, a relatively short incubation period (e.g. ≈ 4 h) should be used for preparing co-cultures. However, the efficiency of macrophages adhesion decreased with a shorter incubation period. We calculated that the efficiency of MDMs and TDMs adhesion for 4 h is around 20%, thus the actual number of macrophages that adhered to the epithelial carpet was much lower than the added number. When taking this adhesion efficiency into consideration, the density of macrophages onto the epithelial carpet in our co-culture models (seeding density: 5 × 10^4^ macrophages/cm^2^) was around 1.0 × 10^4^ macrophages/cm^2^, slightly lower than that in human airway wall (≈3.5 × 10^4^ macrophages/cm^2^) from the lung tissue sections (Grashoff et al., 1997). However, at a seeding number of 2 × 10^4^ macrophages/cm^2^, macrophages may already cover around 70% of the insert membrane (Fig. S5), especially at the centre of the membrane. In order to avoid a too high density of macrophages on the epithelial layer, we increased the seeding density to 5 × 10^4^ macrophages/cm^2^. Still the density of adherent macrophages is lower than in lungs *in vivo*.

After the optimization of the co-culture procedure, the co-culture models showed a stronger inflammatory response (IL-1β, IL-10 and TNF-α) to LPS aerosol compared to the mono-culture (Fig. 7). Similar findings were reported in *in vitro* studies on air pollutants, in which inflammatory responses were increased when macrophages had been added to the epithelial cells (Bauer et al., 2015; Tao & Kobzik, 2002; Wottrich et al., 2004). The macrophage-specific cytokines IL-1β and IL-10 were abundantly produced in our co-culture models, suggesting the activation of macrophages. Importantly, IL-1β and IL-10 only accumulated on the apical side (Fig. 7C and D). It is likely that the tight junction in the co-culture models blocks the passage of the cytokines from the apical to the basolateral side. This lack of passage can be seen as another read-out of proper barrier function. In line with this observation, upon LPS exposure the TNF-α concentration on the apical side of the co-culture models was significantly higher than that in mono-culture model, while the TNF-α level was comparable on the basolateral sides for mono-culture and co-culture models (Fig. 7E and F). This may suggest that TNF-α on the apical side of co-culture models, majorly produced by macrophages in response to LPS aerosol, does not cross the epithelial barrier. Also it indicates that TNF-α on the basolateral side of co-culture models is mainly due to the release from Calu-3 cells as a similar level was observed for the mono-culture model.

The use of MDMs and TDMs in co-cultures was previously compared in terms of their availability, differentiation protocols and donors variations (Chanput et al., 2014; Kooter et al., 2017). We further investigated their morphological changes over the co-culture time period and their response to LPS aerosol. Similar morphological changes of MDMs and TDMs were observed within the 9-day co-culture period. Most of the macrophages remained viable until around D6 of co-culture. During the first 3 days of co-culture some of macrophages seemed to be more active with the developed arrow-like cytoplasm and pseudopodia (Fig. 6A and B). After co-culture for 6 days, macrophages started to detach and float upwards, moving out of focus, which were seen as blurry spots (Fig. S6), indicating the loss of viability. A lack of medium supply due to the tight barrier formed by the epithelial carpet during co-culture for 6 days may explain this loss of viability. It suggests that our co-culture models can be used up to 6 days, while for more prolonged exposures macrophages need to be re-added weekly to ensure characteristics of the co-culture model. Cytokine responses to LPS aerosol varied between the Calu-3 + TDM and Calu-3 + MDM models (Fig. 7). Upon LPS exposure an increased concentration of the anti-inflammatory cytokine IL-10 was detected on the apical side of the Calu-3 + MDM model in keeping with M2 polarization by M-CSF (Mosser & Edwards, 2008). In contrast, no IL-10 was detected in the Calu-3 + TDM model, which was possibly due to the M1 state of PMA-differentiated THP-1 monocytes (Maeβ et al., 2014). In line with previous studies that proposed TNF-α as a marker for M1 macrophages (Mosser & Edwards, 2008), a clear concentration-response relationship in TDMs monocultures (M1 macrophages) was observed at LPS levels ranging from 0.08 to 5.12 μg/cm^2^, while TNF-α was not detectable in MDMs monocultures (M2 macrophages) (Fig. S8). Interestingly, on the apical side of the co-culture models, the Calu-3 + MDM model produced more TNF-α compared to the Calu-3 + TDM model. Interactions between macrophages and epithelial cells might play an essential role in promoting the TNF-α release on the apical side, as reported in several studies(Fizeșan et al., 2019) Ji et al., 2018). However, the mechanism of bidirectional communication underlying inflammatory responses in the current co-culture models is still unclear and needs to be further studied.

Taken together, an optimized Calu-3 + MDM co-culture model was created by allowing 4 h adhesion of macrophages onto the epithelial monolayer with the density of 5 × 10^4^ macrophages/cm^2^. Our model showed epithelial barrier integrity under prolonged ALI culture conditions, as well as an increased sensitivity to LPS aerosol in comparison to the Calu-3 mono-culture and Calu-3 + TDM co-culture models at D1 of co-culture. Therefore, we propose this model, provided weekly seeding of MDMs, to be applied in the prolonged ALI exposure to estimate the hazard of aerosols exposures.

## Declaration of competing interest

The authors declare that they have no known competing financial interests or personal relationships that could have appeared to influence the work reported in this paper.
