# Supplementary material for: Optimization of an air-liquid interface in vitro cell co-culture model to estimate the hazard of aerosol exposures
Source: J Aerosol Sci. 2021 Mar;153:105703. doi: 10.1016/j.jaerosci.2020.105703 (PMC7874005; doi:10.1016/j.jaerosci.2020.105703)
Supplement: Multimedia component 1 [file mmc1.docx]

**Supplementary Material**

**Materials and methods**

**Description of epithelial cell lines**

Cell lines 16HBE and BEAS-2B are immortalized from normal human bronchial epithelial cells and widely used in lung research. The 16HBE cells can form well-defined tight junction with highly organized actin filaments (Ehrhardt et al. 2002) while BEAS-2B cells can form a confluent monolayer faster and exhibit higher homology in gene expression pattern compared to primary cells (Heijink et al. 2010). Calu-3 cells can produce features of differentiated, functional human airway epithelial cells, allowing them to be used for modelling the respiratory epithelial barrier (Grainger et al. 2006). Although the H292 cell line is generated from the salivary gland carcinoma cell, it has been often used as lung cell model to study effects of inhalation exposure on gene expression due to high reactivity to total particulate matter and gas/vapor phase (Courcot et al. 2012; Sekine et al. 2015).

**
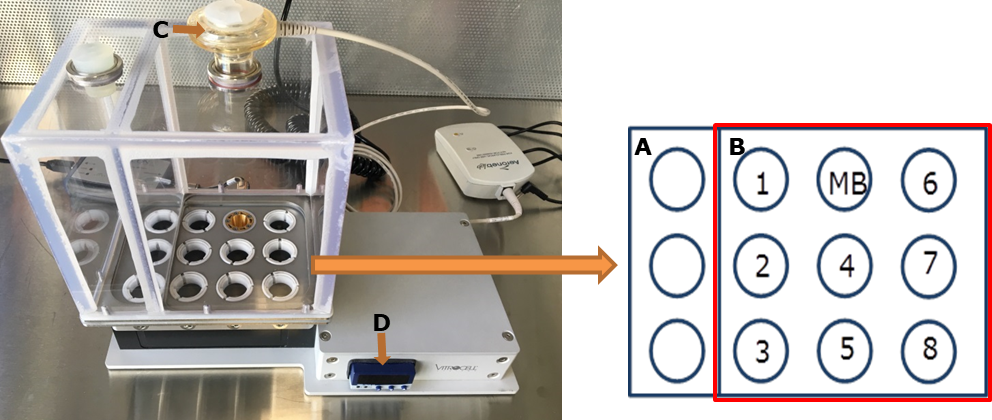
**

**Figure S1.** Air–liquid interface (ALI) Cloud exposure system. A: Chamber for exposure controls, which were not used in this study; B: Chamber for LPS exposure, number 1-8 represents each position, MB represents a microbalance; C: Aerosol nebulizer; D: Temperature controller. More information can be found via https://www.vitrocell.com/inhalation-toxicology/exposure-systems/vitrocell-cloud-system


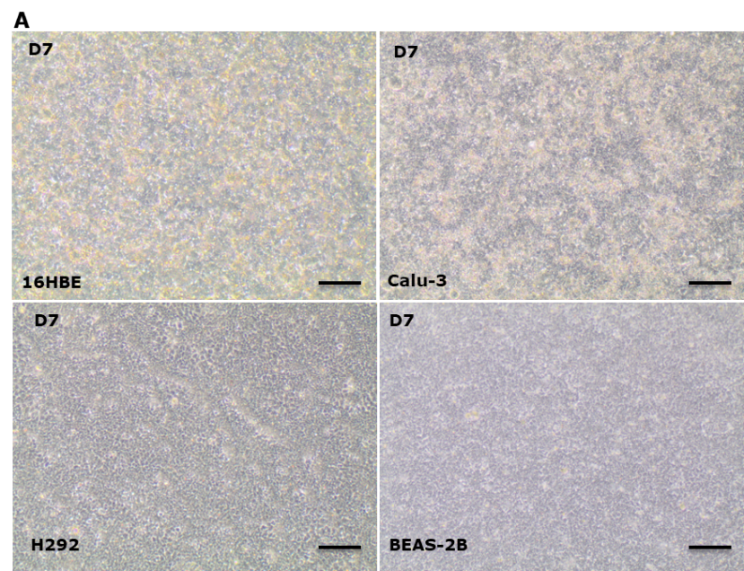

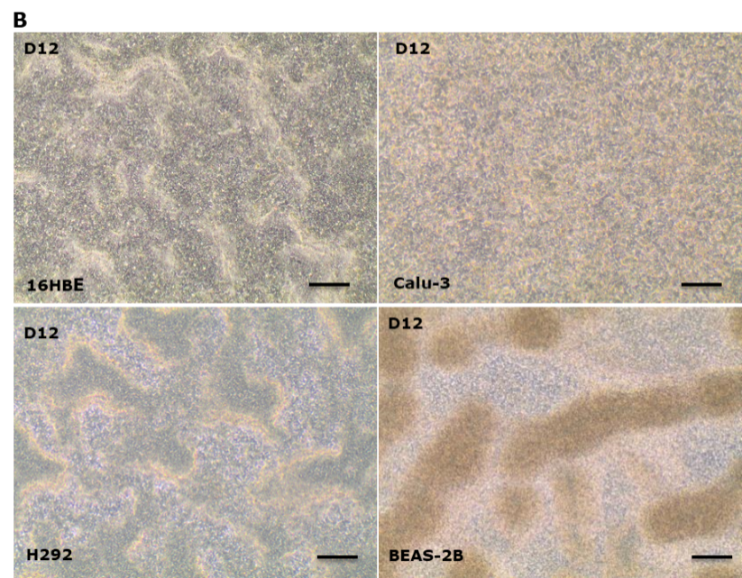

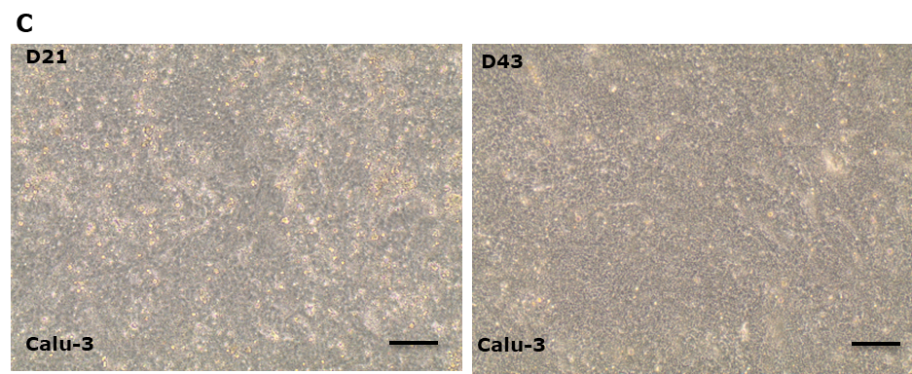


**Figure S2.** Cell morphology of 16HBE, Calu-3, H292 and BEAS-2B cells at day 7 (A) and 12 (B) as well as Calu-3 cells at day 21 and 43 (C). Cells were at submerged culture for 7 days, followed by ALI culture. Scale bars: 100 μm.


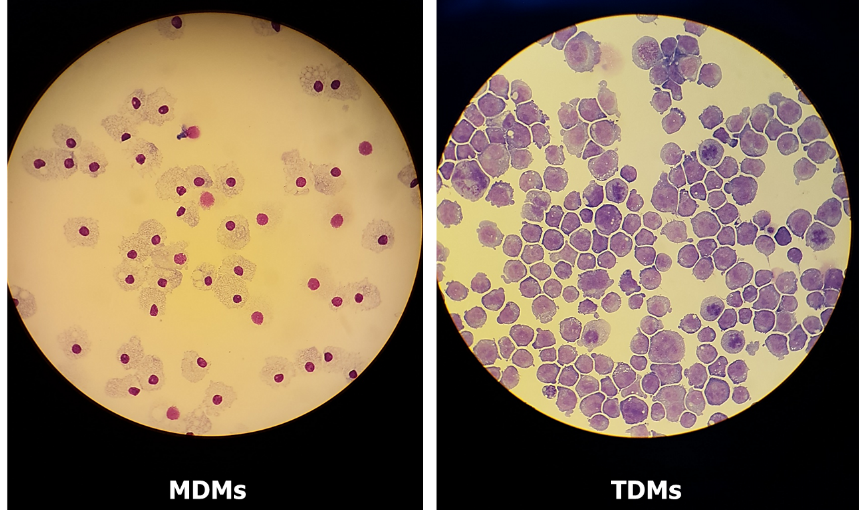


**Figure S3.** May-Grunwald Giemsa stained cytosmears of MDMs (left) and TDMs (right) before adding onto Calu-3 cells.

**A MDM-4hrs**


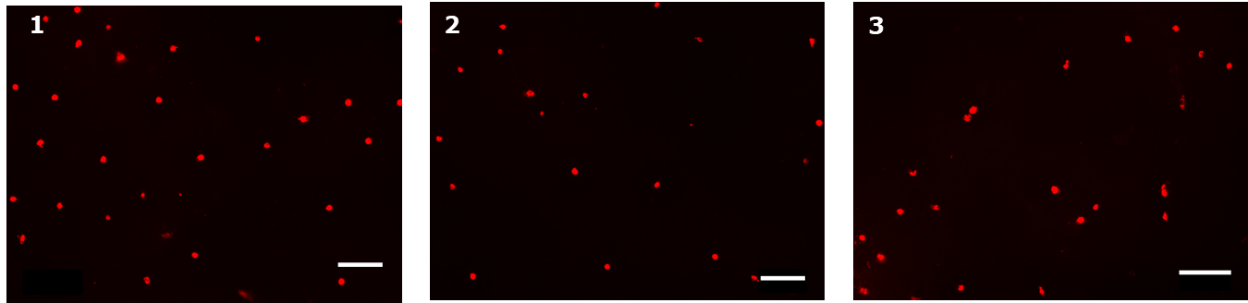


**MDM-24hrs**


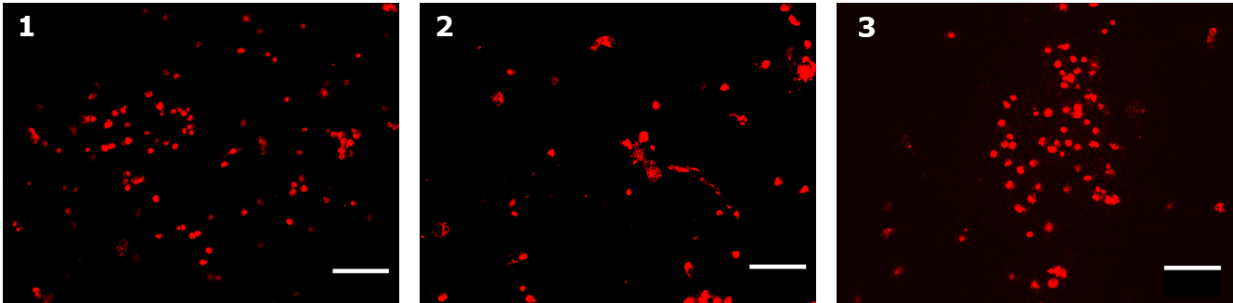


**B TDM-4hrs**


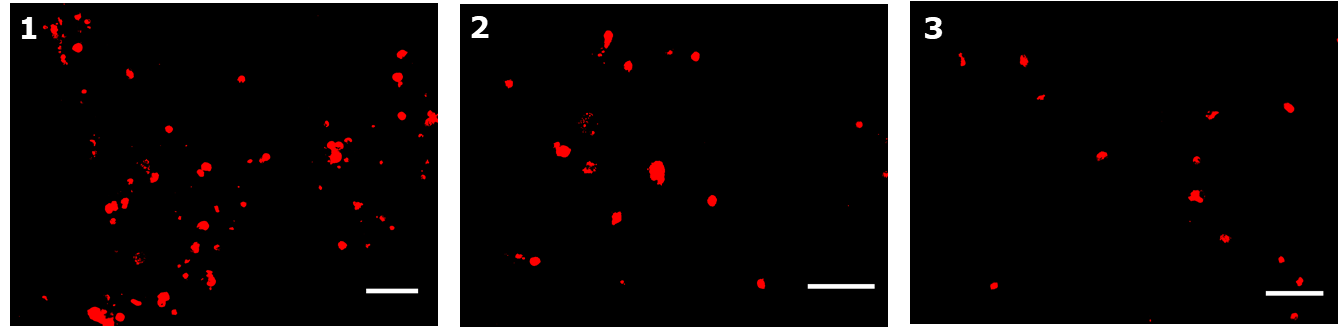


**TDM-24hrs**


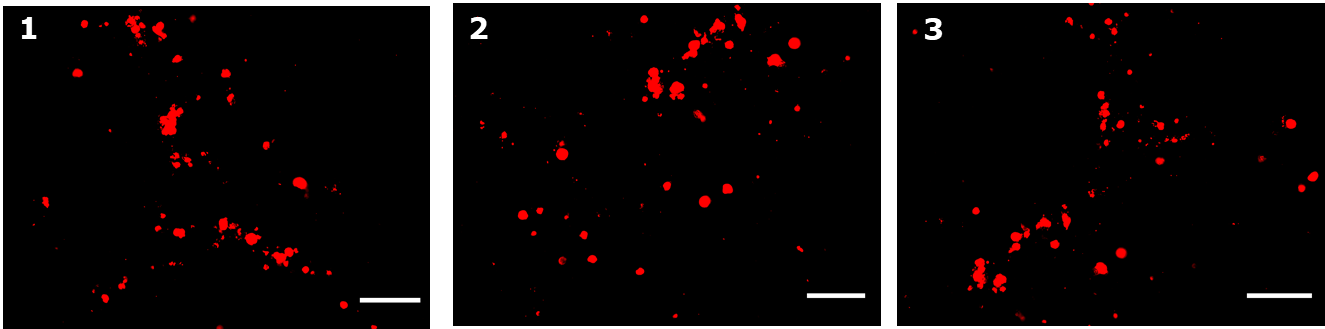


**Figure S4.** Fluorescence microscopy images of MDMs (A) and TDMs (B) after 4 or 24 hrs adhesion onto the Calu-3 epithelial carpet. The seeding density of MDMs and TDMs is 2.0×10^4^ cells/cm^2^. Macrophages were labelled with Vybrant DiI dye in red. Scale bars: 100 μm. Number 1, 2, and 3 represent the images taken at random areas of the membrane.


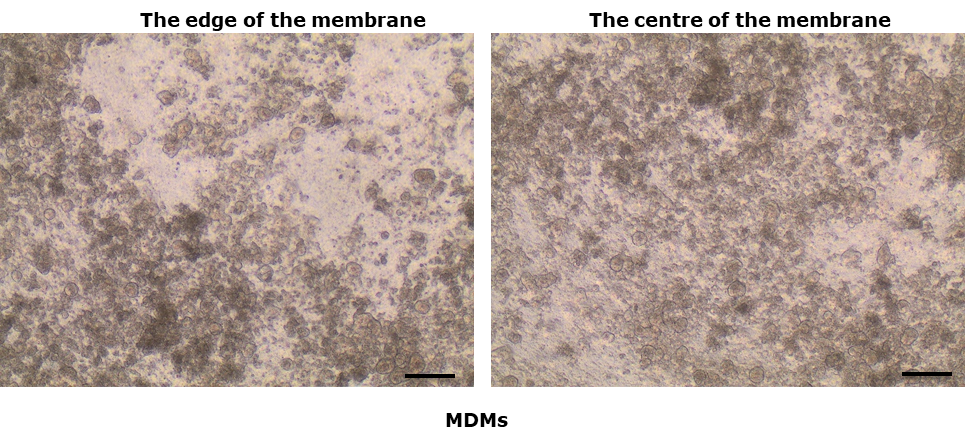


**Figure S5.** Microscopy images of MDMs (density: 2.0×10^4^ cells/cm^2^) after seeding onto the epithelial carpet. Scale bars: 100 μm.

**
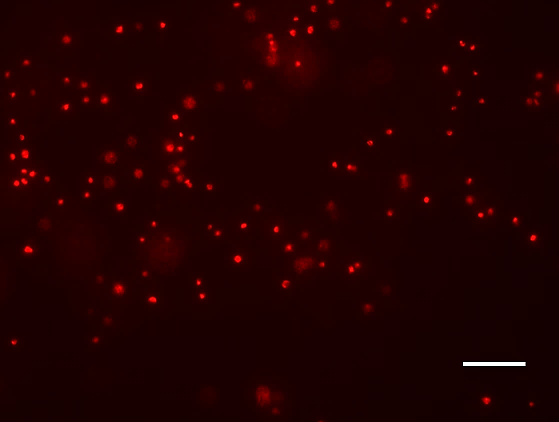
**

**Figure S6.** Fluorescence microscopy images of MDMs (4 hrs adhesion, density: 5.0 × 10^4^ cells/cm^2^) at D6 of co-culture. Scale bars: 200 μm.

**Figure S7.** Effects of exposure to Milli Q water aerosol in co-culture models in 12 well inserts including cell viability, LDH release and the production of IL-8. Error bars indicate the standard deviation of 3 or 4 parallel inserts with cells.

**Figure S8.** TNF-α release from TDMs and MDMs after exposure to LPS at concentrations from 0.08 to 5.12 μg/cm^2^. Error bars indicate the standard deviation of 3 parallel inserts with cells.

**T****able S1.** The density of the Calu-3 cells and the ratio between Calu-3 cells and macrophages (1.0 × 10^4^ macrophages/cm^2^) at Day 0, 1, 3, and 6 for the co-culture. Results for the Calu-3 cells density were obtained from 3 parallel inserts.

| **Co-culture day** | **Day 0** | **Day 1** | **Day 3** | **Day 6** |
| --- | --- | --- | --- | --- |
| Density (Calu-3 cells/cm^2^) | 176421 ± 2389 | 192798 ± 7421 | 207211 ± 16925 | 226842 ± 7421 |
| Ratio  (Calu-3 cells: macrophages) | ≈ 18 | ≈ 19 | ≈ 20 | ≈ 22 |

**References**

Courcot E, Leclerc J, Lafitte J-J, et al. (2012) Xenobiotic metabolism and disposition in human lung cell models: comparison with in vivo expression profiles. Drug Metabolism and Disposition 40(10):1953-1965

Ehrhardt C, Kneuer C, Fiegel J, et al. (2002) Influence of apical fluid volume on the development of functional intercellular junctions in the human epithelial cell line 16HBE14o–: implications for the use of this cell line as an in vitro model for bronchial drug absorption studies. Cell and tissue research 308(3):391-400

Grainger CI, Greenwell LL, Lockley DJ, Martin GP, Forbes B (2006) Culture of Calu-3 cells at the air interface provides a representative model of the airway epithelial barrier. Pharmaceutical research 23(7):1482-1490

Heijink IH, Brandenburg SM, Noordhoek JA, Postma DS, Slebos D-J, van Oosterhout AJ (2010) Characterisation of cell adhesion in airway epithelial cell types using electric cell–substrate impedance sensing. European Respiratory Journal 35(4):894-903

Sekine T, Sakaguchi C, Fukano Y (2015) Investigation by microarray analysis of effects of cigarette design characteristics on gene expression in human lung mucoepidermoid cancer cells NCI-H292 exposed to cigarette smoke. Experimental and Toxicologic Pathology 67(2):143-151
